# Characteristics and experiences of family caregivers in the mental health setting

**DOI:** 10.17843/rpmesp.2022.392.11111

**Published:** 2022-06-30

**Authors:** Claudia P. Cantillo-Medina, Alix Y. Perdomo-Romero, Claudia A. Ramírez-Perdomo

**Affiliations:** 1 Programa de Enfermería, Facultad de Salud, Universidad Surcolombiana, Huila, Colombia. Universidad Surcolombiana Programa de Enfermería Facultad de Salud Universidad Surcolombiana Huila Colombia

**Keywords:** Chronic Illness, Mental Health, Mental Disorders, Caregivers, Nursing Care

## Abstract

**Objective.:**

To describe the characteristics and experiences of family caregivers of persons with mental disorders in a highly complex institution.

**Materials and methods.:**

Mixed method, explanatory study, with an initial quantitative and then qualitative sequence. The quantitative phase was carried out using validated instruments: characterization sheet of the person with chronic illness-family caregiver dyad; the Nkongho Caregiving Ability Inventory (CAI), and the Zarit burden scale. The qualitative phase was conducted with a hermeneutic phenomenological approach; the information was collected through in-depth interviews to understand the needs of the caregivers.

**Results.:**

The caregivers were mostly women, mainly mothers and daughters, home-occupied and self-employed, with basic primary education, low socioeconomic level and low caregiving skill level; a significant number of caregivers perceived some degree of overload. The themes identified in the analysis of the interviews were: feeling fear before the disease; the experience of caregiver fatigue; losing one’s job: another cost of the disease; fear of delegating care; love: a support for the caregiver; needing support to care.

**Conclusions.:**

The caregiver experiences a complex situation associated with the high demand for care, and conditions under which mental health impairment progresses.

## INTRODUCTION

Worldwide, the care of people with mental disorders (MD) is a challenge due to its impact on physical, psychological and social functionality, which leads to a high degree of disability for the individual and a considerable health burden for society ^1^. An illness that lasts more than one year requires continuous medical care and causes limitation of daily living activities ^2^; in addition to a significant emotional and economic cost that affects the quality of life ^1^, related to the lack of social support and the modification in work, family and personal dynamics ^3^.

Budget restrictions in Ibero-America have exacerbated the inequities and lack of funding for home care programs ^4^ for individuals with MD. This has been aggravated by the phenomenon of displacement and the delegation of obligations, where health systems shift formal care from the hospital setting to families ^1^, making this a public and collective health problem ^5^.

With the progression of the MD, the person becomes dependent and requires the support of a caregiver to assist him/her in daily activities and for optimal treatment ^6^. Assuming the role of caregiver causes structural, procedural and emotional changes in the family unit, which can negatively affect the caregiver ^7^ ^,^ ^8^, due to the unpredictability, transitions, complexity and interaction with the ill person ^9^.

The family health of the ill person is affected in the ability to function and adapt to stressful life events ^10^, added to the stigma that produces social rejection due to its association with violence ^11^ and the loss of control during crises, which can endanger his or her life or that of others. Therefore, the caregiver must use various strategies to cope with the emotional instability of his or her family member until he or she can control the situation and recover ^12^.

The caregiver, on most occasions, carries out his or her work in isolation from the social environment, in the solitude of his or her home and in anonymity, without being recognized by health professionals ^13^. Some studies found in the literature address caregiver burden or overload in individuals with MD ^14^ ^-^ ^16^; however, there is a gap in knowledge related to the exploration of variables such as caregiver characteristics, caregiving skill and overload, as well as the identification of caregivers’ needs through the use of a qualitative approach, for an in-depth of the conditions in which the caregiver of individuals with MD lives.

For the reasons stated above, the aim of this study is to describe the characteristics and experiences of the caregivers of family members with MDs in a care center for individuals with MDs in the city of Neiva, Colombia.

**KEY MESSAGES**

Motivation for the study: The lack of knowledge of the characteristics and experiences of family caregivers in the context of mental health prevents the recognition, understanding and visibility of their conditions and needs.

Main findings: The conditions and needs of caregivers are glimpsed through the deepening of their lived experiences; an increase in vulnerability that affects their quality of life is evidenced.

Implications: Educational and psychosocial strategies should be developed, based on public policies that have an impact on the empowerment and well-being of those involved.

## MATERIALS AND METHODS

### Design

Explanatory mixed method study, with an initial quantitative sequence followed by a qualitative phase that was continued with the integration of the results and data during interpretation. A hermeneutic phenomenological approach was used in the qualitative phase.

### Population, sample and type of sampling

The quantitative collection included all caregivers (n=64) of individuals confined in a care center for individuals with MDs, diagnosed as shown in Table 1; the average age 48.6 years. Five caregivers participated in the qualitative phase, who were selected by means of a criterion-oriented casual sampling ^20^.

**Table 1:** Diagnosis of persons with mental disorders (n=64).

Diagnostic	n	%
Organic mental disorders	3	4.7
Mental and behavioral disorders due to psychoactive substances.	9	14.0
Schizophrenia, schizotypal and delusional disorders	18	28.1
Mood or affective disorders	24	37.5
Neurotic, somatoform and stress-related disorders	4	6.3
Behavioral syndromes associated with physiological alterations and physical factors	2	3.1
Personality disorders	4	6.3

### Selection criteria

The following inclusion criteria were taken into account for both approaches: over 18 years of age, engaged in caregiving for a period of more than three months and more than three hours per day, between December 2019 and March 2020 in a care center for individuals with MDs in the city of Neiva, southern Colombia.

### Procedures

Data collection during the quantitative phase was carried out by the researchers and the research assistants, by using online instruments (Google forms), after acceptance of the virtual informed consent.

Qualitative information was collected through in-depth interviews ^21^ conducted by the main researcher. The caregivers were contacted by telephone for the interviews, which lasted approximately one hour and were recorded and transcribed by the research assistants. A field diary was kept to ensure that impressions, ideas and reflections were not lost during the analysis; anonymity was maintained.

### Variables

The sociodemographic variables and characteristics of the caregivers were assessed using the characterization sheet of the person with chronic disease-family caregiver dyad ^17^; caregiving skill using Nkongho’s caregiving ability inventory (CAI) ^18^; and overload using Zarit’s overload scale ^19^. The instruments are described below:

*Characterization sheet of the person with chronic disease-family caregiver dyad*

This instrument has face and content validity for Latin America. It presents 42 items that address the dyad in three categories: general conditions that include the sociodemographic profile; the perception of burden and support, and the means of information and communication available to them ^17^.

*Nkongho’s Caregiving Ability Inventory (CAI).*

This instrument is made up of subscales that respond to three factors associated with understanding oneself and others: knowledge (low score of 76.3 or less, mean of 76.4-83, and high of 84 or more); courage (low score of 62.4 or less, mean of 62.5-73, and high of 74 or more); and patience (low score of 60 or less, mean of 61-65.1, and high of 65.2 or more). The overall rating is considered low when it is 203 or less, mean 203.1-220.2, and high 220.3 or more. This instrument has a Cronbach’s alpha of 0.89 and a stability of r=0.80 in Colombia ^18^.

*Zarit’s overload scale*

This instrument has a Cronbach’s alpha of 0.86 for the Colombian context. It is comprised of 22 items, Likert-type questions with five response options where 1 is “never” and 5 is “almost always”. A score of less than 46 indicates no overload, from 46 to 56 indicates mild overload and greater than 56 indicates intense overload ^19^.

### Data analysis

Data analysis was conducted with IBM SPSS® statistical program, version 22. For categorical variables, we used frequencies and percentages. For interval or ratio variables, the descriptive statistics mean, median, standard deviation, minimum and maximum were used.

The study of the qualitative data was carried out by means of thematic analysis ^22^, which is described as a method to identify, analyze and report common patterns (themes) within the data; a table was constructed in a Word file, where the units of meaning were identified and grouped into common themes. Discussion and reflection were carried out by the researchers to organize the final themes, the connections and interconnections between them. The collection and analysis of the information was carried out at the same time, the results were returned to the participants and feedback was provided by them.

### Ethical criteria

Study approved by the Ethics and Bioethics Committee of the Universidad Surcolombiana by act 007 of 2019, in compliance with the requirements of Resolution 08430 of 1993 based on the ethical principles of autonomy, respect, justice, beneficence, truthfulness, non-maleficence, confidentiality and suitability.

For the qualitative approach, we considered the stringent criteria proposed by Lincoln and Guba ^23^ of credibility, transferability, conformability and reliability. During the reflexivity process ^24^, we recognized the importance of the participation of the researcher and the participant, in an attempt to maintain openness towards the other and trying to see the world in a different way.

## RESULTS

**Figure 1:**
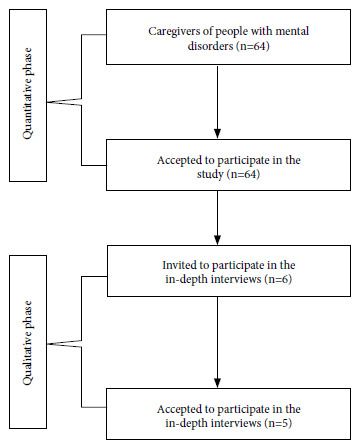
Selection of participants in the quantitative and qualitative phase.

The selection of participants for the quantitative and qualitative phases is presented in Figure 1.

The caregivers were mostly women (67.2%), aged between 27 and 59 years, mainly mothers and daughters, with a stable partner, primary school education, home-occupied and self-employed, with low socioeconomic level, were caregivers with no previous experience, had support from the beginning of the diagnosis, and practiced the Catholic religion (Table 2).

**Table 2:** Sociodemographic characteristics of the caregiver of the person with mental health impairment.

Variable		n	%
Sex	Female	43	67.2
	Male	21	32.8
Age group	Adolescence (12 to 18 years)	1	1.6
	Youth (19 to 26 years)	5	7.8
	Adulthood (27 to 59 years)	39	60.9
	Older adult (60 years and more)	19	29.7
Marital status	Single	17	26.6
	Free union	20	31.2
	Married	15	23.4
	Separated	6	9.4
	Widower	6	9.4
Religion	Catholic	52	81.3
	Christian	10	15.6
	Other religion	2	3.1
Level of religious commitment	Low	9	14.1
	Medium	32	50.0
	High	23	35.9
Area of residence	Rural	6	9.4
	Urban	58	90.6
Schooling	Primary school	21	32.8
	Secondary school	10	15.6
	Middle education	20	31.2
	Labor technique	3	4.7
	Undergraduate	9	14.1
	Postgraduate	1	1.6
Occupation	Home	24	37.5
	Student	4	6.3
	Community leader	2	3.0
	Self-employed	22	34.4
	Employee	11	17.2
	Pensioner	1	1.6
Socioeconomic level	Low	55	85.9
	Medium	9	14.1
Previous care experience	Yes	20	31.2
	No	44	68.8
Experiences in months	3 - 6	7	10.9
	7 - 10	7	10.9
	11 - 14	11	17.2
	Over 14	39	61.0
Care since diagnosis	Yes	42	65.6
	No	22	34.4
Sole caregiver	Yes	25	39.1
	No	39	60.9
Shared care	Yes	39	60.9
	No	25	39.1
Hours of care	1 to 8 hours	20	31.2
	9 to 16 hours	22	34.4
	17 to 24 hours	22	34.4

The caregivers, regarding their caregiving skill, showed a low level of the three dimensions: 50% in knowledge, 64.1% in courage and 75% in patience; we observed the same behavior in the global skill rating with 71.9% (Table 3).

**Table 3:** Results of the Caregiving Ability Inventory (CAI) measurement and degree of overload (n=64).

Variables		n	%
Knowledge	Low	32	50.0
	Medium	16	25.0
	High	16	25.0
Courage	Low	41	64.1
	Medium	16	25.0
	High	7	10.9
Patience	Low	48	75.0
	Medium	16	25.0
Global skill	Low	46	71.9
	Medium	12	18.7
	High	6	9.4
Degree of overload (Zarit)			
Absence of overload		24	37.5
Mild overload		20	31.2
Intense overload		20	31.3

Our results show that 31.2% of caregivers had moderate overload and 31.3% had intense overload (Table 3). 

Five caregivers of individuals with MDs were interviewed. The following themes were reported in the analysis of the interviews:

### Feeling fearful of the disease

When a loved one is diagnosed with a MD, it causes concern and emotional stress due to the stigma attached to this person’s health condition; in addition, they are terrified of changes in medication due to the exacerbation of the symptoms of the disease. Fear of episodes of aggressiveness leads the caregiver to feel “frightened” and uncertain.

“At first, I tried to scare myself because I said no, if that’s how it is as a boyfriend, it’s for life. ... There are people who attack themselves or others, I was afraid that I would arrive and she wouldn’t understand me and I would hurt someone or she would hurt herself. That’s why I got scared” (CMP 1).

“One gets desperate, and thinks, is she going to recover? and how long? we are terrified that they will suspend her medications because the disease will increase, it scares us, that’s why we are waiting for monitoring. We are scared of two things, knowing that she is in this situation and not knowing if she will be able to control (the disease)” (HFC 2).

### The caregiver’s experience of fatigue

The caregiver manifests that he/she performs his/her role with great effort, dedicates a great deal of time to his/her work and, in most cases, expresses physical and emotional exhaustion, as well as social isolation, due to the dependence and instability of his/her family member.

“We went to bed late, she was sick, the child was young and I arrived at work irritated, I have not been a problem, but it is annoying, starting the morning, arriving at work and starting with that burden on top of me, it is a problem, it is a problem... I want to get home to sleep, but I have to take care of her...” (CMP 1).

“It has been complicated for me, because I can’t leave him alone, he takes to the street, I have to keep an eye on him, many times he is very aggressive and rude to me... It was very hard for us, at that time my husband was living, it was very hard for me, very hard, because he was the youngest of the children, so we resigned ourselves to take him with that disease and to support him” (JPM 3).

“That’s like having a gift for people, for me it’s not so difficult, sometimes I get tired, for that I take a medicine and you have to keep going” (MCE 5).

### Losing one’s job: another cost of illness

The commitment of caregivers to provide care to their loved one sometimes leads them to lose their jobs due to the great demands of caregiving, which is related to the amount of time and demand required on a daily basis, taking into account their inability to take care of themselves adequately, in addition to the multiple commitments they must fulfill as members of a family.

“I had to say “I can't work anymore, my wife is sick, I have a small child, 2, 3 years old, it’s over...”. I have quit many jobs because of her, I see that she is sick and I cannot take care of her, so I quit...” (CMP 1).

“You don't think about anything else, you can’t focus, I was working and I had to quit because you only think about the welfare of that person. Leaving my job did not affect me financially... but professionally, I had to stop because my goal was my mother’s recovery” (HFC 2).

“He lives in and out of the hospital for a month, a month and a half, and he comes back and they hand him over to me, and that’s why I haven’t been able to do anything, I get jobs, but I can’t because of him...it’s chaos” (JPM 3).

### The fear of delegating care

For caregivers, delegating care causes fear as they believe that care will not be provided with the same quality and dedication, they feel that only they are prepared to do so, and they are the ones who have known their family member best since the diagnosis of the disease; in addition, they consider themselves responsible for carrying out this task. Another important aspect that produces fear in the caregiver is related to the fear of violence exercised by other family members when they are in charge of caring for the loved one.

“I cannot leave my mother’s care, because if my sisters take care of her, they will beat her, if I get sick, I will have to look for a home to take care of her, my mother told me that if the time came when I could not take care of her, I should leave her under a bridge, so I ask God to have mercy on her and on me” (EPH 5).

“I have been with my son all my life, he began to have the disease when he was 16 years old, he was a very good student, when the disease developed, my husband died and we both continued to fight, I am alone with him, I have a daughter, but she does not love him, she treats him badly, so I live worried, I have no one, I have no resources...” (JPM 3).

### Love: a support for the caregiver

Caregivers assume their role out of love, fulfill all the wishes of the person they care for, devote all their time seeking their well-being, helping them to move forward and being a support to move forward.

“Those people should have people around them who love them, who really want to sacrifice because it is partly a sacrifice, to sacrifice for them, for love... I do all that because I am in love with her, I have not lost that illusion, that it is possible to live with bipolar disorder, it is possible” (CMP 1).

“I thought about positive things and knowing that I was doing it with love because I knew she was my mom, that made me feel better... The truth was that I did it with that love, because she was my mom, I was a little worried about my daughter, but she’s with the dad, I knew she was going to be fine; there I went and said, well I'm going to face the situation, I was going with that positivity” (HFC 2).

“She is a girl who has managed to get ahead because you have to handle them with love and dedication, always supporting them, encouraging them to believe in themselves... They have mood swings, they feel very sad, sometimes a little aggressive, you have to know how to handle them, channel those feelings, supporting them in a positive way with love and tolerance so that they can achieve their goals” (NEC 4).

“Have patience, my mom hits me, and I let her hit me, she grabs me and spanks me, I keep this hand swollen she always hits me there, I just take her hand away and tell her not to be foolish, and I tell her why does she want to hit me? I love her so much” (MCE 5).

### Needing support to care

Care must be shared, the caregiver needs to have recreational spaces, he/she must be very aware of him/herself and know his/her limits in order not to give up. One of the aspects that affect the caregiver is related to the economic impact that the disease has on the family nucleus and especially on the caregiver, they require economic support to meet the needs of the ill person and that are inherent to the disease; however, the illness of their family member leads them to extreme situations due to the impossibility of working and covering expenses in order to support the family.

“My husband did it for my health, I was desperate not being able to do anything for my mom, letting the days go by and not doing anything, 10 months that she was like that in that situation, I always had the support of my husband and my dad, they told me “calm down”” (HFC 2).

“Sometimes I go out, I get very depressed and feel bad, I go to some friends, to an aunt who sometimes helps me with clothes or shoes. I go there or to my sisters and I tell them my problems, although they don’t help me much, they are also poor” (JPM 3).

“I always have support, usually it is a sister, who is also very loving, she likes to share, teach, educate, it is natural to us caregivers of children with mental disabilities, .... I get up and ask God a lot to enlighten me, give me wisdom to be able to help them” (NEC 4).

“My need would be to have a person who would be here with me day and night, so that when I go to sleep she would be watching over me, or that I could say well, I am going to downtown all day today and you can leave with peace of mind, that they give you food, medicine and have money, have money to buy things for you. If there is no money, what can you buy things with?” (EPH 5).

## DISCUSSION

The caregivers are mainly women of productive age, mothers and daughters, with stable partners, low educational level, informal jobs, low socioeconomic level, have support for caregiving and profess the Catholic religion. With low levels in the dimensions of knowledge, courage and patience, as well as in the overall assessment of caregiving skills, they present levels of overload ranging from mild to intense.

The characteristics of the caregivers coincide with those previously described in the literature, the role is mainly assumed by women ^14^ ^,^ ^25^, feminization persists as the main characteristic of formal and informal care ^25^, generally mothers in the cycle of mature adulthood with severe deterioration of their physical health and with mental health risks typical of aging ^15^.

Caregivers showed total dependence ^25^. Most of the caregivers have a low educational and socioeconomic level, are dedicated to their home, since maintaining a working life is complicated by the dedication and time invested in the performance of the caregiving role ^25^, which are unfavorable conditions for well-being ^16^. The circumstances described above increase the vulnerability of families because they impoverish them ^26^ ^,^ ^27^; additionally, they must assume multiple requirements not covered by the health system ^26^.

The caregivers had a partner and a bond with the cared person (children and parents), which are aspects that contribute to overcome the obstacles and challenges imposed by the physical limitations ^25^. Caregivers, most of them with support and without previous experience in the role, care for a time longer than 14 months. They make adjustments to their work obligations ^27^ and develop strategies for coping and adaptation to the disease in search of well-being for the patient and family members ^12^; the whole family group may be affected, and will depend on internal and external support, and on the individual resources of its members ^27^ ^,^ ^28^, there being a strong relationship between the quality of life of the caregivers, the family relationship and the mismatch in the dynamics and the diagnosis ^1^.

Most caregivers expressed that they perceive overload, which may be associated with the multiple responsibilities they assume regarding the tasks required by the degree of dependence, specialized and individualized care ^29^. This generates feelings and emotions of anguish, fear, guilt and worry in the families, added to the lack of knowledge about the management of the health condition, making them feel incapable in their caregiving work ^28^.

Similarly, the received support was rated at a medium level overall by most of the population, and reached the highest level for religious and family support. These are important aspects to strengthen, as suggested by other authors ^28^, with plans that need to be suitable for the needs of individuals and families, taking into account the effect of mental health disorders in the reduction of their quality of life ^10^.

Consequently, caregivers of persons with mental health disorders live in conditions that increase their vulnerability, with the risk of entering the vicious circle of caregiving from the moment they receive the news of their family member’s illness, feeling fear of the disease, due to the recurrence of crises, prolonged dedication to the work ^18^, progression of the situation, and the effects on their lives ^9^. The impact of caregiving can cause a greater effort and uncertainty for those who live with and care for the family member ^3^ ^,^ ^4^ ^,^ ^12^, and shows the experience of caregiver fatigue added to the situation of losing their job, which are aspects that corroborate the quantitative findings related to the presence of overload and the wear and tear of maintaining independent work, threatened by the constant presence of the disease.

Caregivers use love as support and as a coping strategy, by creating practices to provide wellbeing to their family member, showing care with love as a fundamental aspect when assuming the leading role in facing health crises, and always prioritizing the maintenance of the affective bond ^12^. Faced with the need to provide and achieve greater well-being and with the aim of feeling fulfillment and a little peace of mind, caregivers develop spirituality through family prayer, which allows them, despite the pain and fatigue, to maintain the hope that favors the ability to transcend and thus alleviate the emotional, physical and psychological wear and tear ^30^. Needing support to care for and rely on, gives meaning to life, facilitates acceptance of their new situation ^7^, allows social integration, and contributes to the prevention of loneliness ^13^.

The limitations of the study are related to the use of convenience sampling and the small sample size, despite the fact that we used the entire population of caregivers. Regarding the qualitative approach, the design we used to explore the meaning of the phenomenon is a limitation because the results cannot be generalized. 

In conclusion, we can affirm that the care of the individual with MD represents a complex situation associated with the demand for care and the progress of the disorder. Most of the population with MD showed total dependence, and consequently, they live in vulnerable conditions, which ratifies the importance of developing educational and psychosocial strategies that have an impact on their empowerment and well-being.
