# Supplementary material for: Characteristics and experiences of family caregivers in the mental health setting
Source: Rev Peru Med Exp Salud Publica. 2022 Jun 30;39(2):185–92. doi: 10.17843/rpmesp.2022.392.11111 (PMC11397668; doi:10.17843/rpmesp.2022.392.11111)
Supplement: Translation

# Translation: Características y experiencias de los cuidadores familiares en el contexto de la salud mental

**Authors:** Claudia P. Cantillo-Medina, Alix Y. Perdomo-Romero, Claudia A. Ramírez-Perdomo

**Affiliations:** 1 Programa de Enfermería, Facultad de Salud, Universidad Surcolombiana, Huila, Colombia.

**Keywords:** Enfermedad Crónica, Salud Mental, Trastornos Mentales, Cuidadores, Cuidado de Enfermería

## Abstract

**Objetivo.:**

Describir las características y experiencias de los cuidadores familiares de personas con trastorno mental, atendidos en una institución de alta complejidad.

**Materiales y métodos.:**

Estudio de método mixto, explicativo, con una secuencia inicial cuantitativa y luego cualitativa. La fase cuantitativa se realizó mediante instrumentos validados: ficha de caracterización de la díada persona con enfermedad crónica-cuidador familiar; el inventario de habilidad de cuidado (CAI) de Nkongho, y la escala de sobrecarga de Zarit. La fase cualitativa se realizó con un enfoque fenomenológico hermenéutico, la información se recolectó mediante entrevistas en profundidad para comprender las necesidades de los cuidadores.

**Resultados.:**

Los cuidadores fueron, en su mayoría, mujeres, principalmente madres e hijas, con educación básica primaria, ocupación hogar y dedicados al trabajo independiente, nivel socioeconómico bajo, nivel de habilidad de cuidado bajo y un importante número de cuidadores perciben algún grado de sobrecarga. Los temas identificados en el análisis de las entrevistas fueron: sintiendo miedo ante la enfermedad; la experiencia del cansancio del cuidador; perder el trabajo: otro costo de la enfermedad; el temor de delegar el cuidado; el amor: un soporte para el cuidador; necesitando apoyo para cuidar.

**Conclusiones.:**

El cuidador experimenta una situación compleja asociada con la gran demanda de cuidados, y condiciones en que progresa la alteración de la salud mental.

## INTRODUCCIÓN

A nivel mundial el cuidado de las personas con trastornos mentales (TM) es un reto por el impacto en la funcionalidad física, psicológica y social, que conlleva a un alto grado de discapacidad para el individuo y una considerable carga sanitaria para la sociedad ^1^. Una enfermedad que dura más de un año necesita atención médica continua y ocasiona limitación de las actividades de la vida diaria ^2^; además de un importante costo emocional y económico que afecta la calidad de vida ^1^, con relación a la falta de apoyo social y la modificación en las dinámicas laborales, familiares y personales ^3^.

Las restricciones presupuestales en Iberoamérica han exacerbado las inequidades y la falta de financiamiento de los programas de cuidado en el hogar ^4^ para las personas con TM. Esto se ha magnificado por el fenómeno de desplazamiento y la delegación de obligaciones, donde los sistemas de salud derivan los cuidados formales del ámbito hospitalario hacia las familias ^1^, convirtiéndose esto en un problema de salud pública y colectiva ^5^.

Con el progreso de los TM la persona genera dependencia y requiere del soporte de un cuidador para su asistencia en las actividades de la vida cotidiana y para un tratamiento óptimo ^6^. Asumir el papel de cuidador ocasiona cambios estructurales, procesuales y emocionales en la unidad familiar, que puede afectar negativamente al cuidador ^7^ ^,^ ^8^, por la imprevisibilidad, transiciones, complejidad e interacción con la persona enferma ^9^. 

La salud familiar de la persona enferma se afecta en la capacidad de funcionar y adaptarse a acontecimientos vitales estresantes ^10^, sumado al estigma que produce rechazo social por su asociación con la violencia ^11^ ^) ^y por la pérdida de control en las crisis, lo que puede poner en peligro su vida o la de otros. Por lo tanto, el cuidador debe utilizar diversas estrategias para afrontar la inestabilidad emocional de su familiar hasta poder controlar la situación y recuperarlo ^12^.

El cuidador, en la mayoría de las ocasiones, realiza su trabajo en el aislamiento del entorno social, en la soledad de su hogar y en el anonimato, sin ser reconocido por los profesionales de la salud ^13^. Al revisar la literatura se encuentran estudios que abordan la carga o sobrecarga del cuidador en las personas con TM ^14^ ^-^ ^16^; sin embargo, existe un vacío en el conocimiento relacionado con la exploración de variables como las características del cuidador, habilidad del cuidado y la sobrecarga, así como la identificación de las necesidades de los cuidadores mediante el empleo de un enfoque cualitativo, para una comprensión profunda de las condiciones en las que vive el cuidador de las personas con TM.

Por las razones anteriormente expuestas, el objetivo de este estudio es describir las características y experiencias de los cuidadores de familiares con TM, atendidos en un centro de cuidado a personas con TM en la ciudad de Neiva en Colombia.

**MENSAJE CLAVE**

Motivación para realizar el estudio: el desconocimiento de las características y experiencias de los cuidadores familiares en el contexto de la salud mental impide reconocer, comprender y visibilizar sus condiciones y necesidades.

Principales hallazgos: se vislumbran las condiciones y necesidades de los cuidadores a través de la profundización en sus experiencias vividas; se evidencia un aumento de la vulnerabilidad que afecta su calidad de vida.

Implicancias: se deben elaborar estrategias educativas y psicosociales basadas en políticas públicas que impacten en el empoderamiento y bienestar de los involucrados.

## MATERIALES Y MÉTODOS

### Diseño

Estudio de método mixto explicativo, con una secuencia inicial cuantitativa luego una fase cualitativa que se continuó con la integración de los resultados y datos en la interpretación. En la fase cualitativa se empleó un enfoque fenomenológico hermenéutico.

### Población, muestra y tipo de muestreo

En la recolección cuantitativa participaron todos los cuidadores (n=64) de las personas recluidas en un centro de cuidado a personas con TM, diagnosticadas como aparece en la Tabla 1; promedio de edad de 48,6 años. Mientras que en la fase cualitativa participaron cinco cuidadores, seleccionados mediante un muestreo casual orientado por criterio ^20^.

**Tabla 1:** Diagnóstico de las personas con trastornos mentales (n=64).

Diagnóstico	n	%
Trastornos mentales orgánicos	3	4,7
Trastornos mentales y de comportamiento por sustancias psicoactivas	9	14,0
Trastornos de esquizofrenia, esquizotípico y delirios	18	28,1
Trastornos del humor o afectivos	24	37,5
Trastornos neuróticos, somatomorfos y relacionados con el estrés	4	6,3
Síndromes del comportamiento asociados con alteraciones fisiológicas y factores físicos	2	3,1
Trastornos de personalidad	4	6,3

### Criterios de selección

Se tuvieron en cuenta los siguientes criterios de inclusión para los dos enfoques: mayor de 18 años, dedicados al cuidado por un período mayor a tres meses y más de tres horas diarias, atendidos entre diciembre del 2019 y marzo del 2020 en un centro de cuidado a personas con TM en la ciudad de Neiva, al sur de Colombia. 

### Procedimientos

La recolección de la información en la fase cuantitativa fue realizada por las investigadoras y los auxiliares de investigación; se utilizaron instrumentos en línea (formularios de Google), previa aceptación del consentimiento informado virtual.

La información cualitativa se recolectó mediante entrevistas en profundidad ^21^ realizadas por la investigadora principal. Los cuidadores fueron contactados telefónicamente para la realización de las entrevistas, con una duración de una hora, aproximadamente, estas fueron grabadas y transcritas por los auxiliares de investigación. Se llevó un diario de campo para garantizar que las impresiones, ideas y reflexiones no se perdieran durante el análisis, ademas, se mantuvo el anonimato.

### Variables

Se evaluaron las variables sociodemográficas y características de los cuidadores mediante la ficha de caracterización de la díada persona con enfermedad crónica-cuidador familiar ^17^; la habilidad del cuidado mediante el inventario de habilidad de cuidado (CAI) de Nkongho ^18^; y la sobrecarga mediante la escala de sobrecarga de Zarit ^19^. A continuación, se describen los instrumentos: 

*Ficha de caracterización de la díada persona con enfermedad crónica-cuidador familiar*

Este instrumento tiene una validez facial y de contenido para América Latina. Presenta 42 ítems que abordan a la diada en tres categorías: condiciones generales que incluyen el perfil sociodemográfico; la percepción de carga y apoyo, y los medios de información y comunicación de los que disponen ^17^.

*El inventario de habilidad de cuidado (CAI) de Nkongho*

Este instrumento está conformado por las subescalas que responden a tres factores asociados con el entendimiento del propio ser y de otros: conocimiento (calificación baja de 76,3 o menos, media de 76,4-83 y alta de 84 o más); valor (calificación baja de 62,4 o menos, media de 62,5-73 y alta 74 o más) y paciencia (calificación baja de 60 o menos, media de 61-65,1 y alta de 65,2 o más). La calificación global se considera como baja cuando tiene 203 o menos, media de 203,1-220,2, y alta de 220,3 o más. Este instrumento tiene un alfa de Cronbach de 0,89 y una estabilidad de r=0,80 en Colombia ^18^.

*La escala de sobrecarga de Zarit*

Este instrumento tiene un alfa de Cronbach de 0,86 para el contexto colombiano. Está compuesto por 22 ítems, preguntas de tipo Likert con cinco opciones de respuesta donde 1 es «nunca» y 5 es «casi siempre». Un puntaje menor de 46 indica no sobrecarga; de 46 a 56 sobrecarga leve; mayor de 56 indica una sobrecarga intensa ^19^.

### Análisis de los datos

El análisis de la información se realizó con el programa estadístico IBM SPSS®, versión 22. Para las variables categóricas se emplearon frecuencias y porcentajes. Para las variables de intervalo o razón se aplicaron los estadísticos descriptivos media, mediana, desviación estándar, mínimo y máximo. 

El estudio de los datos cualitativos se realizó mediante el análisis temático ^22^, el cual se describe como un método para identificar, analizar y reportar patrones comunes (temas) dentro de los datos; se elaboró una tabla en Word donde se identificaron las unidades de significado que fueron agrupadas en temas comunes. Se realizó discusión y reflexión por parte de las investigadoras para organizar los temas finales, las conexiones e interconexiones entre ellas. La recolección y el análisis de la información se realizó a la par, los resultados fueron devueltos a los participantes, y retroalimentados por ellos.

### Criterios éticos

Estudio aprobado por el Comité de Ética y Bioética de la Universidad Surcolombiana mediante acta 007 del 2019, en cumplimiento de los requerimientos de la Resolución 08430 de 1993 fundamentados en los principios éticos de autonomía, respeto, justicia, beneficencia, veracidad, no maleficencia, confidencialidad e idoneidad.

En el enfoque cualitativo se tuvieron en cuenta los criterios de rigor propuestos por Lincoln y Guba ^23^ de credibilidad, transferibilidad, conformabilidad y confiabilidad. En el proceso de reflexividad^ (^ ^24^ se reconoció la importancia de la participación del investigador y el participante, en un intento de mantener una apertura hacia el otro y procurando ver el mundo de una manera diferente.

## RESULTADOS

La selección de los participantes en la fase cuantitativa y cualitativa se presenta en la Figura 1.

**Figura 1:** Selección de participantes fase cuantitativa y cualitativa.

Los cuidadores fueron, en su mayoría, mujeres (67,2%), en edades entre 27 a 59 años, principalmente madres e hijas, con pareja estable, educación básica primaria, ocupación hogar y dedicados al trabajo independiente, nivel socioeconómico bajo, cuidan sin experiencia anterior, tenían apoyo desde el inicio del diagnóstico, y practicaban la religión católica (Tabla 2).

**Tabla 2:** Características sociodemográficas del/la cuidador/a de la persona con alteración de la salud mental.

Variable		n	%
Sexo	Femenino	43	67,2
	Masculino	21	32,8
Grupo etario	Adolescencia (12 a 18 años)	1	1,6
	Juventud (19 a 26 años)	5	7,8
	Adultez (27 a 59 años)	39	60,9
	Adulto mayor (60 años y más)	19	29,7
Estado civil	Soltero	17	26,6
	Unión libre	20	31,2
	Casado	15	23,4
	Separado	6	9,4
	Viudo	6	9,4
Religión	Católica	52	81,3
	Cristiana	10	15,6
	Otra religión	2	3,1
Nivel de compromiso religioso	Bajo	9	14,1
	Medio	32	50,0
	Alto	23	35,9
Área de residencia	Rural	6	9,4
	Urbano	58	90,6
Escolaridad	Básica primaria	21	32,8
	Educación secundaria	10	15,6
	Educación media	20	31,2
	Técnico laboral	3	4,7
	Pregrado	9	14,1
	Posgrado	1	1,6
Ocupación	Hogar	24	37,5
	Estudiante	4	6,3
	Líder comunitario	2	3,0
	Trabajador independiente	22	34,4
	Empleado	11	17,2
	Pensionado	1	1,6
Nivel socioeconómico	Bajo	55	85,9
	Medio	9	14,1
Experiencia anterior en el cuidado	Sí	20	31,2
	No	44	68,8
Experiencias en meses	3 - 6	7	10,9
	7 - 10	7	10,9
	11 - 14	11	17,2
	Más de 14	39	61,0
Cuidado desde diagnóstico	Sí	42	65,6
	No	22	34,4
Único cuidador	Sí	25	39,1
	No	39	60,9
Cuidado compartido	Sí	39	60,9
	No	25	39,1
Horas de cuidado	1 a 8 horas	20	31,2
	9 a 16 horas	22	34,4
	17 a 24 horas	22	34,4

Los cuidadores, en relación con la habilidad de cuidado, presentaron en las tres dimensiones un nivel bajo: conocimiento en 50%, valor en 64,1% y paciencia en 75%, igual comportamiento se observó en la calificación de la habilidad global con 71,9% (Tabla 3).

**Tabla 3:** Resultados de la medición del inventario habilidad de cuidado (CAI) y grado de sobrecarga, (n=64).

Variables		n	%
Conocimiento	Bajo	32	50,0
	Medio	16	25,0
	Alto	16	25,0
Valor	Bajo	41	64,1
	Medio	16	25,0
	Alto	7	10,9
Paciencia	Bajo	48	75,0
	Medio	16	25,0
Habilidad global	Bajo	46	71,9
	Medio	12	18,7
	Alto	6	9,4
Grado de sobrecarga (Zarit)			
Ausencia de sobrecarga		24	37,5
Sobrecarga leve		20	31,2
Sobrecarga intensa		20	31,3

Los cuidadores presentaron sobrecarga moderada en un 31,2% y sobrecarga intensa en un 31,3% (Tabla 3). 

Se entrevistaron a cinco cuidadores de personas con TM. En el análisis de las entrevistas se reportaron los siguientes temas: 

### Sintiendo miedo ante la enfermedad

El diagnóstico de TM de un ser querido provoca preocupación y estrés emocional ante el estigma que se tiene por la condición de salud de esta persona; además, ellos se sienten aterrados ante los cambios en la medicación por la exacerbación de los síntomas de la enfermedad. El temor ante los episodios de agresividad lleva al cuidador a sentir «susto» e incertidumbre. 

"Al principio traté de asustarme porque yo dije no, si eso es así como novio es para toda la vida. … Hay personas que se agreden o agreden a otras, a mi me daba miedo llegar y que ella no me entendiera y, le hiciera daño a alguien o se hiciera daño ella. Por eso me asusté (CMP 1).

«Uno se desespera, y piensa ¿será que se va a recuperar? y ¿cuánto tiempo?, nos aterra que le suspenden los medicamentos porque se incrementa la enfermedad, nos da susto, por eso estamos pendientes del control. Nos asustan dos cosas, saber que ella se encuentra en esta situación y no saber si ella se pudiera controlar (la enfermedad)» (HFC 2).

### La experiencia del cansancio del cuidador

El cuidador manifiesta realizar su rol con mucho esfuerzo, dedicar gran cantidad del tiempo del día a su labor y, en la mayoría de las oportunidades expresa desgaste físico y emocional, así como aislamiento social, por la dependencia e inestabilidad de su familiar.

“Entonces uno a veces llega y tiene los problemas en el trabajo y la encuentra así toda elevada… Nos acostábamos tarde, ella enferma, el niño estaba pequeño y yo llegaba irritado al trabajo, no he sido de problemas, sino que es molesto, empezar la mañana, llegar al trabajo y empezar con esa carga encima, es un problema, es un problema… Quiero llegar a la casa a dormir, pero toca estar pendiente de ella…” (CMP1).

"Se me ha complicado, porque no lo puedo dejar solo, él coge la calle, tengo que estar pendiente de él, muchas veces es muy agresivo y grosero conmigo… A nosotros nos dio muy duro, en ese entonces vivía mi esposo, a mí me dio muy duro, muy duro, porque era el menor de los hijos, entonces nos resignamos a llevarlo con esa enfermedad y a apoyarlo” (JPM 3).

“Eso es como tener don de gente, para mí no es tan difícil, a veces me canso, para eso me tomo un medicamento y hay que seguir” (MCE 5).

### Perder el trabajo: otro costo de la enfermedad

El compromiso de los cuidadores para brindar cuidado a su ser querido los lleva, en algunas oportunidades, a perder el trabajo por la gran exigencia que conlleva la labor de cuidar, con relación a la cantidad de tiempo y demanda requerida durante el día a día, teniendo en cuenta la incapacidad que tienen para cuidarse de manera adecuada, además de los múltiples compromisos que deben cumplir como miembros de una familia.

“Me tocaba decir “No puedo laborar más, mi mujer está enferma, tengo un niño pequeño, de 2, 3 años, se acabó…”. He renunciado a muchos trabajos por ella, veo que está enferma y no la puedo cuidar, entonces renuncio…” (CMP1).

"No piensas en nada más, no te centras, estaba trabajando y me tocó dejarlo porque solo piensas en el bienestar de esa persona. Dejar el trabajo, en lo económico no me afectaba… sí en lo profesional, un bajón de nota me tocó frenar, porque el objetivo era la recuperación de mi mamá” (HFC2).

“Él vive entrando y saliendo del hospital dura un mes, mes y medio, y vuelve y me lo entregan, y por eso no he podido hacer nada, me salen trabajos, pero no puedo por él…es un caos" (JPM 3).

### El temor de delegar el cuidado

Para quienes cuidan, delegar el cuidado causa temor al creer que los cuidados no serán brindados con la calidad y entrega que ellos lo hacen, sienten que solamente ellos se encuentran preparados para hacerlo, y son quienes más conocen a su familiar desde el diagnosticó de la enfermedad; además, se consideran los responsables para llevar a cabo dicha tarea. Otro aspecto importante que produce temor en el cuidador se relaciona con el miedo a la violencia ejercida por otros familiares cuando se encargan de cuidar al ser amado.

“El cuidado de mi mamá no lo puedo dejar, porque si mis hermanas la llegan a cuidar, le pegan, si me llego a enfermar, me tocará buscar un hogar que la cuide, mi mamá me decía, que si llegaba el momento que no la pudiera cuidar la dejara debajo de un puente, entonces le pido a Dios que tenga misericordia de ella y de mi" (EPH 5).

“He estado toda la vida con mi hijo, a él le empezó la enfermedad desde que tenía 16 años, era muy buen estudiante, cuando se desarrolló la enfermedad, mi esposo murió y seguimos los dos luchando, quede sola con él, tengo una hija, pero no me lo quiere, lo trata mal, entonces vivo preocupada, me le mezquina la comidita, no tengo a nadie, no tengo recursos…" (JPM 3).

### El amor: un soporte para el cuidador

Los cuidadores asumen su rol por amor, cumplen todos los deseos de la persona a quienes cuidan, dedican todo su tiempo, buscando su bienestar, ayudándolos a seguir adelante, siendo un soporte para seguir adelante. 

"Esas personas deben tener alrededor de ellas quienes las quieran, que de verdad se quieran sacrificar porque en parte es un sacrificio, sacrificarse por ellas, por amor… hago todo eso porque estoy enamorado de ella, no he perdido esa ilusión, de que se puede vivir con trastorno bipolar, se puede" (CMP1).

“Pensaba en cosas positivas y en saber que lo hacía con amor porque sabía que era mi mamá, eso me hacía sentir mejor… La verdad lo hacía con ese amor, pues era mi mamá, un poquito me preocupaba mi hija, pero ahí con el papá, sabía que iba a estar bien; allá iba y decía, bueno le voy a hacer frente a la situación iba con ese positivismo” (HFC2).

“Es una niña que ha logrado salir adelante porque hay que manejarlos con mucho amor y dedicación siempre apoyándolos, impulsándolos a lograr a creer en ellos mismos… Tienen cambios de ánimo, sienten mucha tristeza a veces un poco agresivos, hay que saber manejarlos, canalizarles esos sentimientos apoyándolos de manera 

positiva con amor y tolerancia para que puedan lograr sus metas” (NEC4).

"Tener paciencia, mi mamá me pega, y me dejo pegar, ella me agarra y me da palmadas, mantengo esta mano hinchada ella siempre me pega ahí, solo le quito la mano y le digo no sea necia, y le digo que ¿por qué me quiere pegar? Si la quiero tanto" (MCE 5).

### Necesitando apoyo para cuidar

El cuidado debe ser compartido, el cuidador necesita contar con espacios de esparcimiento, debe estar muy pendiente de sí y conocer sus límites para no claudicar. Uno de los aspectos que afectan al cuidador se relaciona con el impacto económico que la enfermedad produce en el núcleo familiar y en especial al cuidador, ellos requieren apoyo económico para suplir las necesidades de la persona enferma y que son propias de la enfermedad, sin embargo, la enfermedad de su familiar los lleva a situaciones extremas por la imposibilidad de trabajar y tener ingresos para sus gastos y sostener a la familia. 

"Mi esposo lo hacía por mi salud, yo me desesperaba al no poder hacer nada por mi mamá, al dejar pasar los días y no hacer nada, 10 meses que ella estuvo así en esa situación, siempre conté con el apoyo de mi esposo y mi papá, me decían “tranquila”" (HFC2).

“A veces salgo, me deprimo mucho y me pongo mal, me voy para donde unas amigas, para donde una tía que a veces me colabora con ropa o con zapatos. Me voy para allá o también donde mis hermanas y les cuento mis problemas, aunque ellas poco me ayudan, ellas también son pobres” (JPM 3).

“Siempre tengo apoyo, por lo general es una hermana, que también es muy amorosa, le gusta compartir, enseñar, educar, nos nace a nosotras las cuidadoras de niños con discapacidades mentales, …. me levanto y le pido mucho a Dios que me ilumine, me de sabiduría para poderles ayudar” (NEC4).

“Mi necesidad sería que tuviera una persona que estuviera día y noche acá conmigo, para cuando me duerma ella estuviera pendiente, o que pueda decir bueno me voy hoy todo el día para el centro y uno se va tranquilo, que le den la comida, los medicamentos y tener plata, tener uno plata para comprarle las cosas, si no hay plata con qué se le compra” (EPH 5).

## DISCUSIÓN

Los cuidadores, principalmente son mujeres en edad productiva, madres e hijas, con pareja estable, bajo nivel escolar, realizan trabajos informales, tienen un nivel socioeconómico bajo, cuentan con apoyo para el cuidado y profesan la religión católica. Con niveles bajos de las dimensiones de conocimiento, valor y paciencia, así como en la valoración global de la habilidad del cuidado; presentan niveles de sobrecarga entre leve e intensa.

Las características de los cuidadores coinciden con lo descrito en la literatura, el papel principalmente lo asumen las mujeres ^14^ ^,^ ^25^, persiste la feminización como característica principal del cuidado formal e informal ^25^, generalmente madres y en el ciclo de adultez madura identificada por presentar mayores grados de deterioro en su salud física y en riesgos de su salud mental propios del envejecimiento ^15^.

Las personas cuidadas presentaron dependencia total ^25^. La mayoría de los cuidadores tienen bajo nivel educativo y socioeconómico, dedicados al hogar, pues el mantenimiento de una vida laboral se complica por la dedicación y tiempo invertido al desempeño del papel de cuidador ^25^, condiciones desfavorables relacionadas con el bienestar ^16^. Las circunstancias anteriormente descritas incrementan la vulnerabilidad de las familias pues las empobrecen ^26^ ^,^ ^27^; adicionalmente, deben asumir múltiples requerimientos no cubiertos por el sistema de salud ^26^.

Los cuidadores contaban con pareja y un vínculo con la persona cuidada (hijos y padres), aspectos que contribuyen a superar los obstáculos y desafíos impuestos por las limitaciones físicas ^25^, los cuidadores, la mayoría con apoyo, sin experiencia anterior para desempeñar el papel, cuidan por un tiempo mayor de 14 meses. Ellos hacen ajustes a sus obligaciones laborales ^27^ y desarrollan estrategias para el afrontamiento y adaptación a la enfermedad en busca de bienestar para el paciente y los miembros de la familia ^12^; todo el grupo familiar puede afectarse, y dependerá del apoyo interno, externo, y de los recursos individuales de quienes la conforman ^27^ ^,^ ^28^, existiendo una fuerte relación entre la calidad de vida de los cuidadores, la relación familiar y el desajuste en la dinámica y el diagnóstico ^1^.

La mayoría de los cuidadores expresaron percibir sobrecarga, la que puede estar asociada con las múltiples responsabilidades que asumen en relación con las tareas que demanda el grado de dependencia, atención especializada e individualizada ^29^, y generan en las familias sentimientos, emociones cargadas de angustia, miedo, culpa y preocupación, sumado al desconocimiento sobre el manejo de la condición de salud, haciéndolos sentir incapaces en su labor de cuidado ^28^.

De igual manera, para la mayoría de la población la satisfacción con los apoyos recibidos fue calificados de manera global en nivel medio, alcanzando el nivel máximo en alto el apoyo religioso y familiar; importantes aspectos para fortalecer como lo plantean los autores ^28^ con planes adecuados a las necesidades de las personas y familias, teniendo en cuenta el efecto de las alteraciones de la salud mental en la disminución de su calidad de vida ^10^.

En consecuencia, los cuidadores de personas con alteraciones de la salud mental viven en condiciones que aumentan su vulnerabilidad, con riesgo de entrar en el círculo vicioso del cuidado desde el momento de recibir la noticia de la enfermedad de su familiar, sintiendo miedo ante la enfermedad, por la recurrencia de las crisis, la dedicación prolongada a la labor ^18^, progresión de la situación, y los efectos de esta en sus vidas ^9^. El impacto de cuidar puede ocasionar un mayor esfuerzo e incertidumbre para quien convive y atiende al familiar ^3^ ^,^ ^4^ ^,^ ^12^, y da cuenta de la experiencia del cansancio del cuidador sumado a la situación de perder el trabajo, aspectos que corroboran los hallazgos cuantitativos relacionados con la presencia de sobrecarga y el desgaste de mantener el trabajo independiente, amenazado por la presencia constante de la enfermedad.

El amor es un soporte para el cuidador como estrategia de afrontamiento, se inventa prácticas para producir bienestar en su familiar, manifiesta cuidar con amor como aspecto fundamental al asumir el protagonismo de enfrentar las crisis de salud priorizando siempre mantener el vínculo afectivo ^12^. Ante la necesidad de proporcionar y lograr un mayor bienestar, con el objetivo de sentir plenitud, y un poco de tranquilidad, desarrolla su espiritualidad por medio de la oración en familia, que le permite, a pesar del dolor y la fatiga, mantener la esperanza que favorece la capacidad de trascender y así aliviar el desgaste emocional, físico y psicológico ^30^. Necesitando apoyo para cuidar y contar con él, da sentido a la vida, facilita la aceptación a su nueva situación ^7^, permite la integración social, y aporta a la prevención de la soledad ^13^.

Las limitaciones del estudio se relacionan con el empleo de un muestreo por conveniencia y el pequeño tamaño de muestra, a pesar de que se tomó toda la población de cuidadores. En el enfoque cualitativo, la limitación se relaciona con el diseño mediante el cual se explora el significado de un fenómeno, por lo que no es posible la generalización de los resultados de la investigación. 

Como conclusión, se puede afirmar que el cuidado de la persona con TM representa una situación compleja asociada con la demanda de cuidados, y progreso del trastorno. La mayor población con TM evidenció dependencia total, en consecuencia, viven en condiciones vulnerables, lo cual ratifica la importancia de desarrollar estrategias educativas y psicosociales, que impacten en el empoderamiento y bienestar.
